# 48491 Development of Marketing Materials for the NJACTS Integrating Special Populations Core

**DOI:** 10.1017/cts.2021.544

**Published:** 2021-03-30

**Authors:** Jessica Zhu, Natalia L. Herman

**Affiliations:** 1 Rutgers University - Ernest Mario School of Pharmacy; 2 Rutgers University

## Abstract

ABSTRACT IMPACT: The development of marketing materials such as flyers and brochures will ultimately be used to promote integration of special populations who are traditionally underrepresented into research by informing and attracting scholars and investigators of available consultative and analytic services that are provided by the ISP Core staff. OBJECTIVES/GOALS: The development of informative and memorable marketing materials is to increase awareness of the ISP Core and its service functions to help with the integration of special populations, as well as promoting scholar and investigator use of these services. METHODS/STUDY POPULATION: After assessing how many CTSA hubs market their ISP services, a flyer and brochure were developed using Adobe InDesign to include information commonly found on CTSA hubs. Flyers and brochures were chosen because they make information physically available outside of a website and be sent to email listservs, making it possible to reach more scholars and investigators. The marketing materials will contain sections to explain the purpose of NJACTS and the ISP Core, list related special populations and available service functions, introduce the ISP Core leadership team, provide examples of past consulting work and contact information for investigators to request service consultations. Flyers will be emailed digitally to listservs and distributed physically along with printed tri-fold brochures to investigators. RESULTS/ANTICIPATED RESULTS: The primary anticipated result from the development of marketing materials include an increased awareness and utilization of ISP Core services and an increased inclusion of special populations in research with NJACTS. The impact of these marketing materials maybe assessed by providing investigators with a short survey when ISP services are requested, which will ask how investigators learned about ISP and its services. DISCUSSION/SIGNIFICANCE OF FINDINGS: Through the work of creating physical marketing materials, the ISP Core will have a method to effectively distribute information about its services, ultimately promoting investigators at all stages to integrate special populations into their research.

